# Subcortical White Matter Changes with Normal Aging Detected by Multi-Shot High Resolution Diffusion Tensor Imaging

**DOI:** 10.1371/journal.pone.0157533

**Published:** 2016-06-22

**Authors:** Sheng Xie, Zhe Zhang, Feiyan Chang, Yishi Wang, Zhenxia Zhang, Zhenyu Zhou, Hua Guo

**Affiliations:** 1 Department of Radiology, China-Japan Friendship Hospital, Beijing, China; 2 Center for Biomedical Imaging Research, Department of Biomedical Engineering, Tsinghua University, Beijing, China; 3 GE Healthcare China, Beijing, China; Institute of Psychology, Chinese Academy of Sciences, CHINA

## Abstract

Subcortical white matter builds neural connections between cortical and subcortical regions and constitutes the basis of neural networks. It plays a very important role in normal brain function. Various studies have shown that white matter deteriorates with aging. However, due to the limited spatial resolution provided by traditional diffusion imaging techniques, microstructural information from subcortical white matter with normal aging has not been comprehensively assessed. This study aims to investigate the deterioration effect with aging in the subcortical white matter and provide a baseline standard for pathological disorder diagnosis. We apply our newly developed multi-shot high resolution diffusion tensor imaging, using self-feeding multiplexed sensitivity-encoding, to measure subcortical white matter changes in regions of interest of healthy persons with a wide age range. Results show significant fractional anisotropy decline and radial diffusivity increasing with age, especially in the anterior part of the brain. We also find that subcortical white matter has more prominent changes than white matter close to the central brain. The observed changes in the subcortical white matter may be indicative of a mild demyelination and a loss of myelinated axons, which may contribute to normal age-related functional decline.

## Introduction

Diffusion tensor imaging (DTI) has been widely used to investigate microstructural deterioration associated with aging, including axonal disintegration and neuron cell loss in white matter (WM) tracts, before morphological changes can be reliably detected by anatomical magnetic resonance imaging (MRI) techniques [1–13]. Compared with morphological measurements, DTI shows increased sensitivity for detecting age-related microstructural alternations [14] in WM. Various DTI studies have shown that fractional anisotropy (FA) decreases and mean diffusivity (MD) increases in WM with normal aging [7–11, 15, 16]. These observations can be assumed to reflect age-related changes in the composition and microstructural information of WM [5, 6].

Subcortical WM contains pyramidal dendrites and short U fibers, which make up around half of the brain’s WM volume in normal persons. It builds the neural connections between cortical and subcortical regions, and constitutes the basis of neural networks for motor, sensory, cognitive and behavioral integration. Therefore, it plays a very important role in normal brain function and any damage or deterioration will affect the basic operation of the related sub-systems.

Like the commonly known WM tracts, subcortical WM may also develop cerebral alterations with aging. For traditional diffusion weighted imaging (DWI) techniques, spatial resolution, typically around 2×2 mm^2^ (in-plane), is quite limited since it is usually provided by single-shot echo planar imaging (EPI) acquisitions. In addition, it has been reported that at such resolutions, partial volume effect will affect quantitative precision [12]. Therefore, it can be very challenging to precisely distinguish subcortical WM from surrounding tissues, and the overwhelming partial volume effect may significantly hamper the investigation of age-related effects in the subcortical WM [17]. To address this issue, Bhagat et al. investigated subcortical WM in both young and elderly groups by reducing the CSF contamination through an inversion recovery technique, although they still used low resolution imaging using 96×128 sampling matrix [18]. They found lower FA values in the subcortical WM in the gyri and genu in the anterior region, but not in the posterior region, when comparing elderly with young normal subjects. As the image resolution used was limited, the accuracy might be compromised. Furthermore, the relatively small cohort and narrow age range limit this study’s applicability as a reference standard. Considering the importance of subcortical WM, it is worthy of further investigation using advanced state of the art DWI techniques, which can provide higher imaging accuracy technically. Compared with the extensive research in the relationship between WM alternations and normal aging, our knowledge of subcortical WM needs to be improved. In addition, one previous study examined the difference between distal and central fiber samples for age-related declines in FA and increases in diffusivity [19]. They reported the changes to be more pronounced in distal regions of the brain than the central part. Since subcortical WM belongs to distal segments of WM, whether there is even more prominent deterioration effect with aging in subcortical WM needs to be determined.

By using multi-shot based techniques, high resolution DTI becomes feasible for detecting more detailed information than commonly used low resolution techniques. It also has advantages over traditional DTI: higher resolution, less blurring and image distortion, as well as suppression of the partial volume effect [20, 21]. Previous studies have shown that on clinical scanners it can even be used to measure diffusion anisotropy information of gray matter, which cannot been performed in traditional measurements [22–24]. There are several different implementations for high resolution DTI, such as multi-shot radial, propeller, spiral and EPI based techniques [25]. Multi-shot EPI has fewer distortion artifacts than the traditional single-shot EPI DWI due to increased sampling bandwidth along the phase encoding direction, and more importantly, EPI based methods have a relatively higher acquisition efficiency than other techniques, thus they have been well accepted.

For subcortical WM, high resolution imaging is desired in order to distinguish it from surrounding tissue structures. In this study, we applied our newly developed multi-shot high resolution DTI technique, self-feeding MUSE (SF-MUSE)[26], to measure subcortical WM changes in healthy persons with a wide age range. Our hypothesis is that with the new detection capability, tissue deterioration can be reliably monitored in the subcortical WM. In addition, since the distal and central fibers change differently with aging [19], we further hypothesize that subcortical WM will display more pronounced changes than WM close to the central brain, and this can be detected. The information obtained here can serve as complementary to previous WM studies in aging. Such an investigation in normal adults may serve as a baseline standard against which patients with pathological disorders can be compared.

## Methods

### Subjects

A total of 53 healthy Asian volunteers were recruited in this study, 47.2% females, ranging from 24 to 79 years of age, with an average age of 54±16 years. Inclusion criteria were: subjects had no history of neurologic, psychiatric, cognitive or behavioral abnormalities. Exclusion criteria included the following: history of neurologic injury or surgery, claustrophobia, and other diseases. Additionally, subjects with deep and periventricular WM abnormalities as detected by their FLAIR T2-weighted images were excluded. Informed consent was obtained from each subject prior to enrollment. The study was approved by the Ethics Committee of the Tsinghua University School of Medicine.

### MRI Data Acquisition

All scans were performed on a GE 3.0T MRI scanner (MR 750, GE Healthcare, Milwaukee, USA) using an 8-channel head coil. A 4-shot single-echo diffusion weighted EPI sequence without navigators was developed and optimized for the DTI acquisitions. One baseline acquisition (b = 0) and 6-direction DWI with NEX = 2 at a b factor of 800 s/mm^2^ were acquired after high order shimming. The remaining imaging parameters were: in-plane resolution: 0.94×0.94 mm^2^, TR/TE = 4000/51ms, FOV = 240×240mm^2^, sampling matrix = 256×256, slice thickness = 4mm, 34 axial slices with no slice gap, and the total scan time was 228 seconds. No cardiac triggering was used during the data sampling due to its reduced scanning efficiency. Instead, data rejection was employed to suppress the severe blood pulsation (see Image Reconstruction section). Additionally, anatomical FLAIR T2 weighted images were also acquired to evaluate potential WM lesions in the subjects. The acquisition parameters were: TR = 9000ms, TE = 170.5ms, TI = 2263ms, FOV = 240×240mm^2^, slice thickness = 5mm, slice gap = 2.0mm, sampling matrix = 256×192, NEX = 1. If lesions were present, the subject was excluded from the study. During the MRI data acquisitions, subjects were told to stay still. In order to further minimize motion, subjects' heads were restrained in the head coil using extendable foam pads.

### Image Reconstruction

The DTI images were reconstructed offline using a self-feeding multiplexed sensitivity-encoding (SF-MUSE) method [26] MUSE [27] is a self-navigating technique for high resolution multishot DTI, providing higher signal sampling efficiency than navigated acquisition schemes. MUSE utilizes SENSE to solve the phase of each excitation and perform the phase correction. Developed from MUSE, SF-MUSE optimizes the phase computation by using prior information regularized SENSE [28]. More importantly, retrospective motion detection and data rejection strategies are performed to exclude unusable data corrupted by severe pulsatile motions, which are common in each subject. If corrupted data is used directly in the final reconstruction, it will introduce image artifacts and errors to the DTI metrics, especially in the mid-brain region. The data rejection threshold was set to 10% as in the original paper. This new SF-MUSE approach can provide more reliable reconstruction with fewer artifacts compared with the original MUSE method. Thus SF-MUSE is a robust method for high resolution diffusion imaging and suitable for practical applications with high throughput. Other technical details can be found in the original paper [26].

### Data analysis

The DTI images were first interpolated from matrix size 256×256 to 512×512 such that ROI can be drawn more easily than in the original images. Diffusion metrics, including FA, radial diffusivity (RD) were then calculated from the DTI images after affine registration using DTI studio [29].

**Fig 1:**
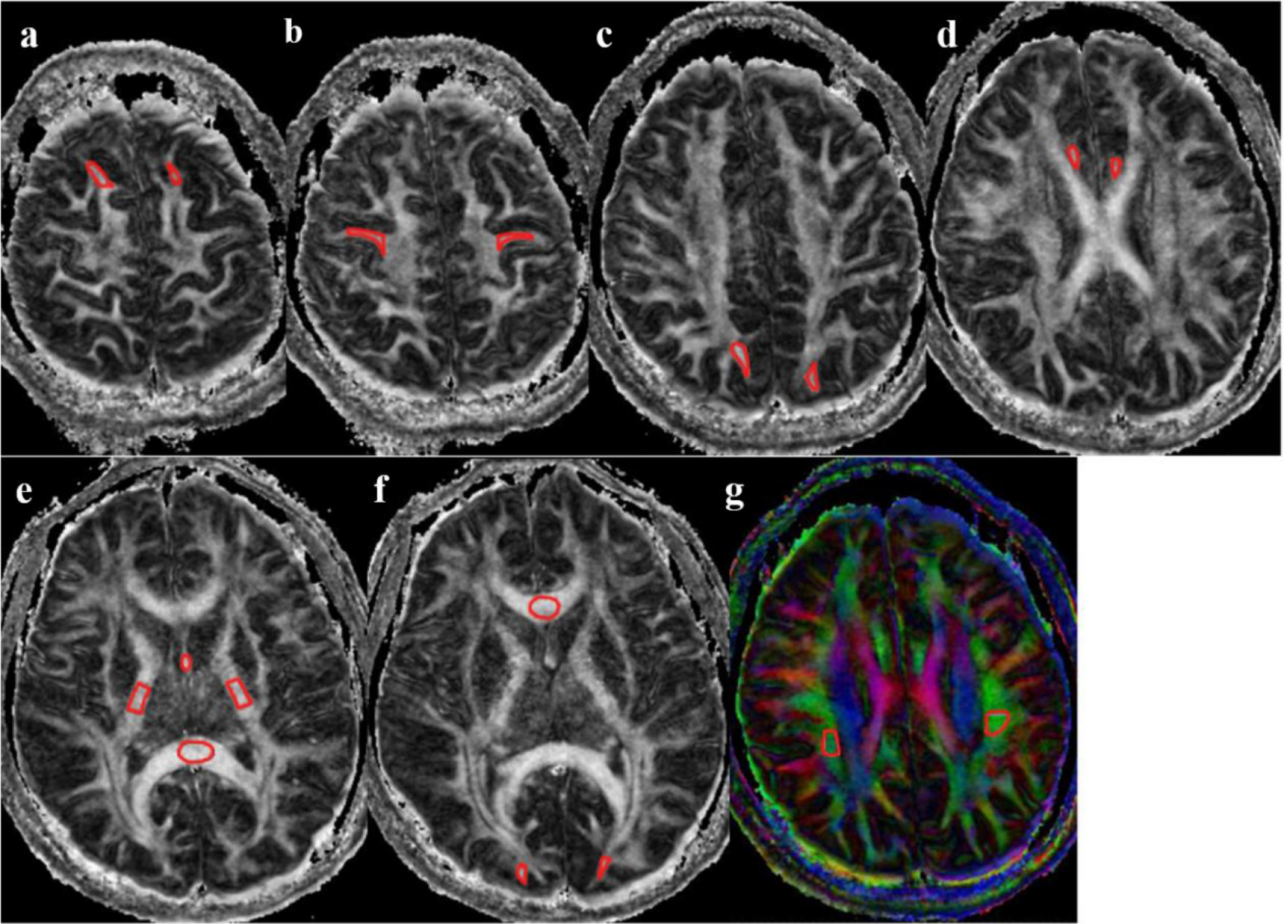
Illustration of ROI drawing. a) WM of bilateral superior frontal gyri; b) WM of bilateral precentral gyri; c) WM of bilateral superior parietal gyri; d) bilateral anterior cingulum; e) fornix, internal capsule and splenium of the corpus callosum; f) genu of the corpus callosum, and WM bilateral occipital; g) superior longitudinal fasciculus.

Irregular regions of interest (ROI) were drawn manually on the subcortical WM in zoomed-in regions by one of the authors, an experienced radiologist with more than 10-years of experience (S.X.). She was blinded to patients’ age and sex. The ROIs included subcortical WM of bilateral superior frontal gyri, bilateral precentral gyri, bilateral superior parietal lobes, bilateral occipital lobes, bilateral anterior cingulum and the fornix. They also included WM ROIs: genu of the corpus callosum, splenium of the corpus callosum (CC), internal capsule (IC), superior longitudinal fasciculus (SLF) (Fig 1). Intense attention was taken to match the borders of cortico-medullary junction within the ROIs and to ensure consistency of ROI locations across the subjects. ROIs were prescribed according to the following criteria: a) Using reconstructed coronal images as references, WM of gyri perpendicular to the axial plane were chosen as much as possible. WM of superior frontal gyri, precentral gyri and superior parietal lobule can be included with this criterion. Irregular ROIs were drawn on the last stage of the branches in the subcortical WM, but not too close to the cortex in order to avoid the partial volume effect. b) The ROIs were quasi-circular on the tangent plane for the cingulum and the fornix, and a maximum FA was be used from multiple slices. c) Because of the varied directionality of the calcarine cortices, subcortical WM with maximum FA values was selected. d) The middle slices in the corpus callosum and in the posterior limb of internal capsule were selected. e) ROIs in the longitudinal fasciculus of the parietal were selected on the colored FA maps. Slices with maximum FA values showing the longitudinal fasciculus were used. A set of example ROIs drawn based on these principles are demonstrated in Fig 1.

In order to ensure reproducibility of the analysis, intra-rating was measured by the same radiologist (S.X.) who re-drew each ROI on 5 sets of the DTI images after 4 weeks. Additionally, in order to assess the consistence of ROI drawing across subjects, another radiologist (Z.Z.) also drew the ROIs on 7 sets of the DTI images. Reliability of the ROI measures was then assessed by Lin’s concordance correlation coefficient (CCC) [30]. A CCC value of 1 indicates perfect agreement; a value <0.5 is considered to be poor agreement, values between 0.5 and 0.7 to be moderate agreement, and values >0.7 to be good to excellent agreement.

As previous studies have used both linear and quadratic regression models for assessing the age-related diffusion indices, we also applied these two methods for the ROI data in this study. The fitting formulae for the linear and quadratic regression models are

$$FA=a_{0}+a_{1}\;*\;age$$ (1)

$$RD=b_{0}+b_{1}\;*\;age$$ (2)

$$FA=c_{0}+c_{1}\;*\;age+c_{2}\;*\;age^{2}$$ (3)

$$RD=d_{0}+d_{1}\;*\;age+d_{2}\;*\;age^{2}$$ (4)

where a0, b0, c0 and d0 are constants, and a1, b1, c1, c2, and d1, d2 are the regression coefficients. For the final model selection, if the 95% confidence interval for the quadratic coefficient in the quadratic model contains 0, then the linear model is accepted. Otherwise, the linear model is not suitable, and the quadratic model is used. If the linear model was selected, an F test was used to evaluate if the slope of the fitted line was less than zero.

## Results

**Fig 2:**
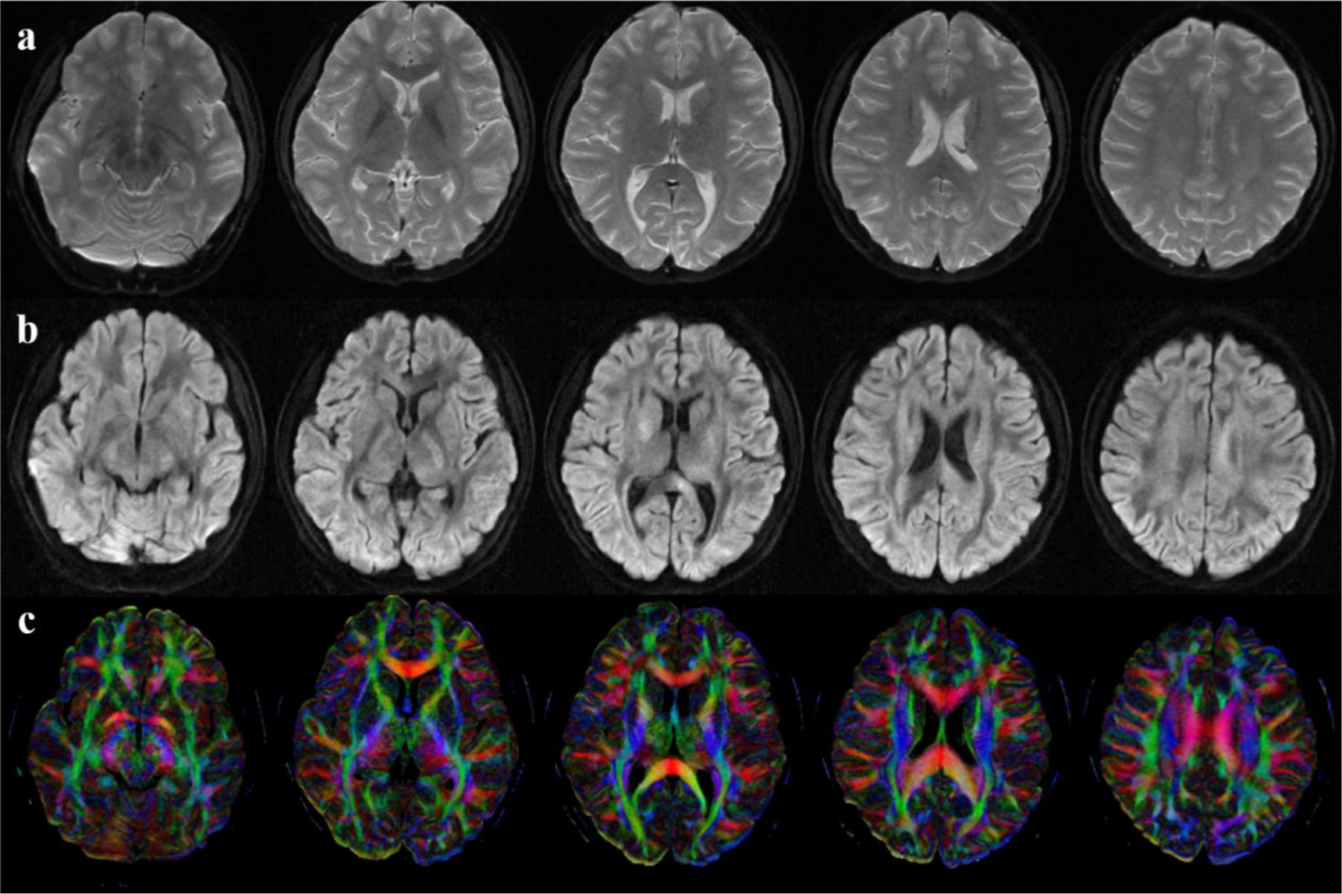
DWI and FA from a representative subject. a) b = 0 images; b) DWI images; c) FA maps. Five representative slices are shown from the left to the right.

Data were successfully obtained from all participants. After images were reconstructed, motion was visually examined. No bulk motion was found in these subjects. Fig 2 demonstrates typical image quality acquired by this 4-shot EPI DTI sequence and reconstructed by the SF-MUSE technique. Compared with traditional single-shot EPI DTI with low resolution (not shown here), higher resolution imaging provides higher resolvability, especially in the subcortical WM. During the reconstruction, the average k-space data rejection rate was 4.09% ±3.67% for all subjects and contamination from corrupt data was well controlled. Different regions showed different rejection rates. For example, in the layers or slices around the brain stem and cerebellum the rate was about 7.79% on average, and about 5.75% around the ventricles, while in the layers near the parietal lobe the rate was lower, 0.30%~2.83%. Such high rejection rates cannot be ignored otherwise errors would be introduced in the resultant data. Therefore data rejection is necessary.

For the reproducibility test in the intra-rating using 7 subjects, the mean CCCs were 0.87 and 0.86 for FA and RD, respectively, which indicates very good agreement in the repeated ROI outlines. For the consistence test of ROI drawing across subjects in the inter-rating, the mean CCCs were 0.83 and 0.65 for FA and RD, respectively, which also gives moderate to good agreement between different raters.

**Fig 3:**
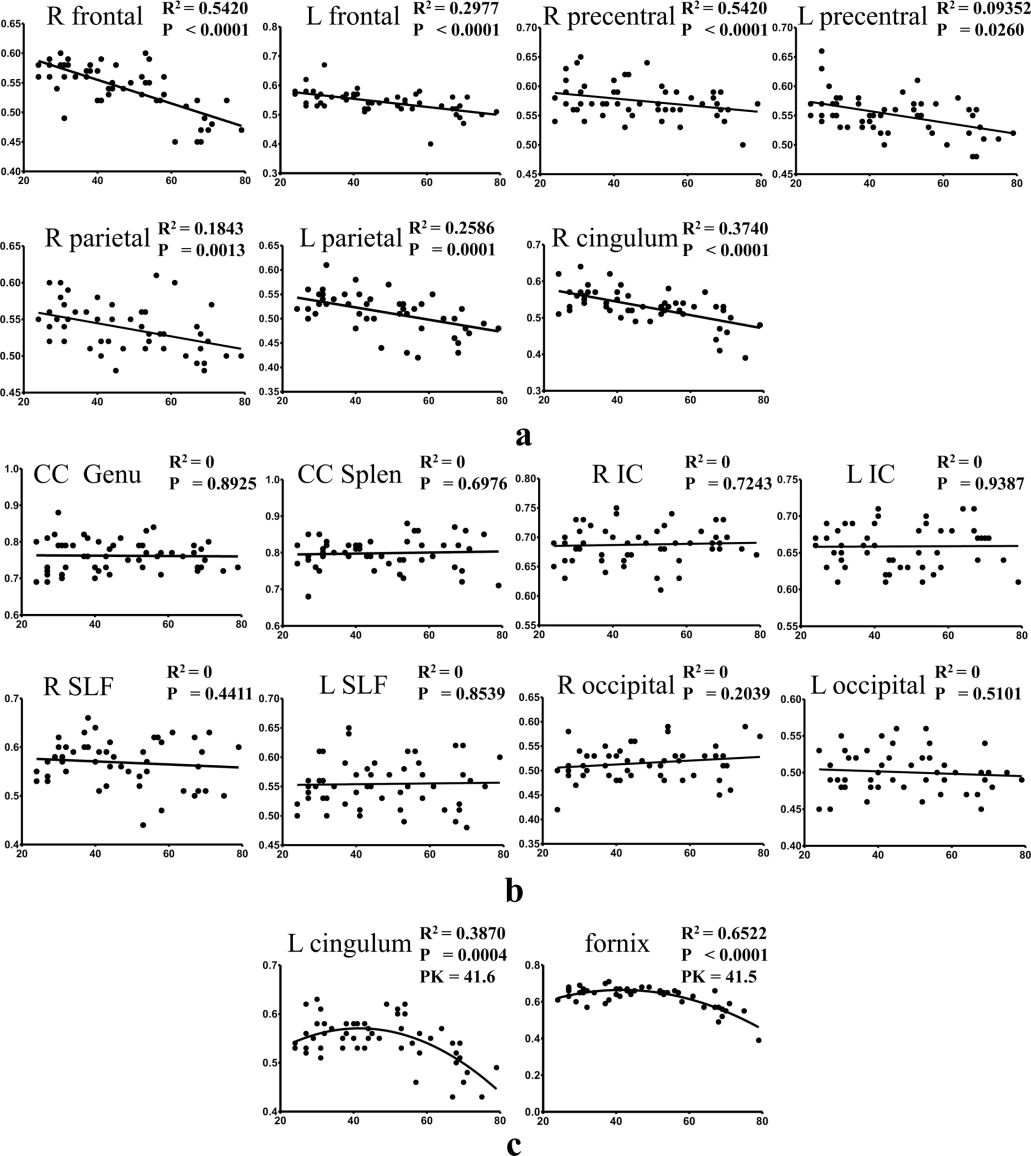
Scatter plots and regression lines for FA as a function of age. y-axis is for FA values while x-axis is for age. a) Regression with negative slopes; b) Regression with zero slopes; c) Regression using quadratic models. P values in a) and b) are for testing the hypothesis whether slope = 0; P values in c) are for testing the hypothesis whether linear model is better than quadratic model; PK in c) represents peak positions of the quadratic fit.

Table 1 shows the model selection and the slope assessment for all ROI data. For the FA values in the selected ROIs, calculation showed that only the left cingulum and the fornix had a better fitting using the quadratic model, while all other ROIs fit better in the linear model. Note that Slope < 0.0 in Table 1 means that the slope of fitted curve is less than zero, which can be illustrated by Fig 3. Similarly, “Slope = 0.0” means the slope of the fitted curve is zero. For these two cases, the preferred model is the linear model. For the RD values, the linear model fit better in the WM of bilateral precentral gyri, genu of the corpus callosum, bilateral internal capsules, left superior longitudinal fasciculus and right occipital. The remaining ROIs had a better fitting using the quadratic model. The goodness of fit for FA and RD values is listed in Table 1 and Table 2, respectively. Multiple comparison correction using Bonferroni correction was applied. Since we conducted linear/quadratic regression in 17 ROIs, a p value < 0.05/17 = 0.0029 was considered to be statistically significant. Besides the fornix, the WM of right frontal gyrus showed the best fit, with R^2^ = 0.542 and 0.432 for FA and RD respectively. The variation was the biggest in the calcarine cortices as it is the most difficult region to measure due to the inconsistent direction of the subcortical WM.

**Table 1:** Statistics of effects of age on regional whiter matter ROIs across the age span for FA.

Test of linearity of effects of age on FA			Characterization of the linear model slope			
Location	95% confidence intervals	Preferred model	P value	Linear model slope	Preferred model	R^2^
R frontal	-7.2E-5 to 1.0E-6	Linear	< 0.0001	-2.00E-03	Slope < 0.0	0.54
L frontal	-3.7E-5 to 5.0E-5	Linear	< 0.0001	-1.39E-03	Slope < 0.0	0.30
R precentral	-5.0E-5 to 2.4E-5	Linear	0.026	-5.81E-04	Slope < 0.0	0.09
L precentral	-4.1E-5 to 3.8E-5	Linear	0.0007	-9.74E-04	Slope < 0.0	0.20
R parietal	-4.1E-5 to 3.6E-5	Linear	0.0013	-8.94E-04	Slope < 0.0	0.18
L parietal	-6.0E-5 to 2.7E-5	Linear	0.0001	-1.26E-03	Slope < 0.0	0.26
R cingulum	-8.3E-5 to 1.2E-5	Linear	< 0.0001	-1.83E-03	Slope < 0.0	0.37
L cingulum	-1.4E-4 to -4.2E-5	Quadratic				0.39
CC genu	-9.4E-5 to 1.6E-5	Linear	0.893	-5.20E-05	Slope = 0.0	0.00
CC splenium	-8.2E-5 to 2.6E-5	Linear	0.698	1.45E-04	Slope = 0.0	0.00
R IC	-3.9E-5 to 4.2E-5	Linear	0.724	9.76E-05	Slope = 0.0	0.00
L IC	-3.2E-5 to 4.2E-5	Linear	0.939	1.95E-05	Slope = 0.0	0.00
Fornix	-1.9E-4 to -9.9E-5	Quadratic				0.65
R SLF	-7.5E-5 to 4.4E-5	Linear	0.441	-3.17E-04	Slope = 0.0	0.01
L SLF	-6.8E-5 to 3.7E-5	Linear	0.854	6.68E-05	Slope = 0.0	0.00
R occipital	-5.5E-5 to 3.6E-5	Linear	0.204	3.97E-04	Slope = 0.0	0.03
L occipital	-6.9E-5 to 1.8E-6	Linear	0.510	-1.66E-04	Slope = 0.0	0.01

**Table 2:** Statistics of effects of age on regional whiter matter ROIs across the age span for RD.

Test of linearity of effects of age on RD			Characterization of the linear model slope			
Location	95% confidence intervals	Preferred model	P value	Linear Model Slope	Preferred model	R^2^
R frontal	2.7E-4 to 1.1E-3	Quadratic				0.43
L frontal	5.5E-5 to 9.4E-4	Quadratic				0.22
R precentral	-1.3E-4 to 7.1E-4	Linear	0.516	-1.90E-03	Slope = 0.0	0.01
L precentral	-2.6E-4 to 6.3E-4	Linear	0.066	5.70E-03	Slope = 0.0	0.06
R parietal	1.8E-4 to 9.9E-4	Quadratic				0.23
L parietal	1.0E-5 to 1.0E-3	Quadratic				0.23
R cingulum	1.3E-4 to 1.3E-3	Quadratic				0.30
L cingulum	4.2E-4 to 1.6E-3	Quadratic				0.33
CC genu	-2.4E-4 to 1.2E-3	Linear	0.986	9.04E-05	Slope = 0.0	0.00
CC splenium	1.2E-4 to 1.4E-3	Quadratic				0.10
R IC	-2.9E-4 to 7.3E-4	Linear	0.075	-6.42E-03	Slope = 0.0	0.06
L IC	-1.3E-4 to 6.1E-4	Linear	0.161	-3.67E-03	Slope = 0.0	0.04
Fornix	2.1E-3 to 3.9E-3	Quadratic				0.66
R SLF	1.3E-5 to 1.1E-3	Quadratic				0.09
L SLF	1.3E-4 to 7.9E-4	Linear	0.378	2.84E-03	Slope = 0.0	0.02
R occipital	-2.3E-4 to 6.0E-4	Linear	0.394	-2.46E-03	Slope = 0.0	0.01
L occipital	1.3E-4 to 9.6E-4	Quadratic				0.17

FA values in the WM of bilateral superior frontal gyri, left precentral gyrus, bilateral superior parietal lobes, and bilateral anterior cingulum showed significant negative slopes or drops from linear regression. FA negatively correlated with age in these regions. The WM of right superior frontal gyrus showed the quickest drop among all regions. Its slope was -0.002, meaning the value dropped 0.02 in one decade. All slope values are listed in Table 1. The quadratic regression shows concave patterns with an apex at around 41 years old in the fornix and left cingulum (Fig 3).

**Fig 4:**
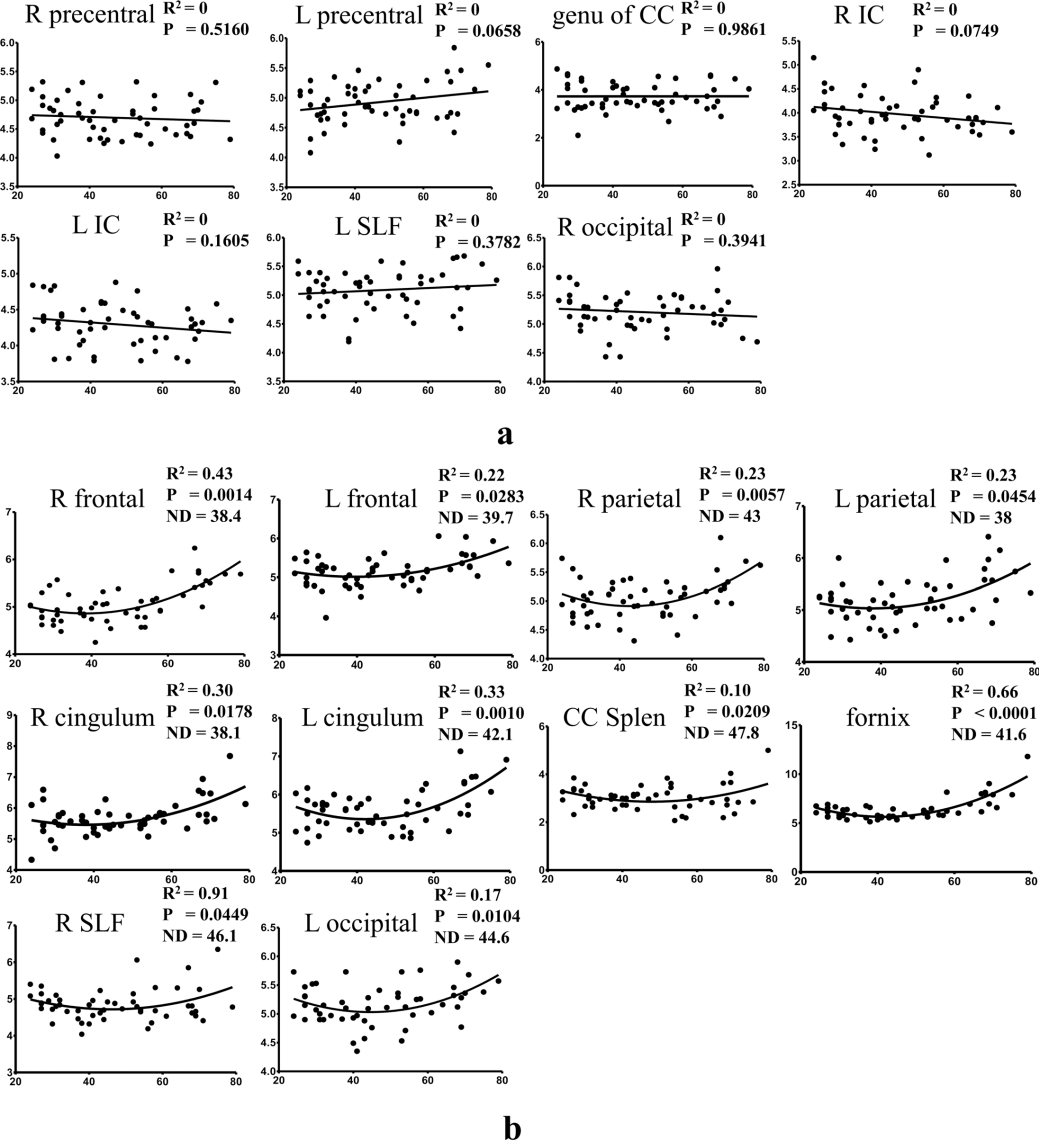
Scatter plots and regression lines for RD as a function of age. y-axis is for RD values while x-axis is for age. a) Regression using linear models; b) Regression using quadratic models. P values in a) are for testing the hypothesis whether slope = 0; P values in b) is for testing the hypothesis whether linear model is better than quadratic model; ND in b) represents nadir position of the quadratic fit.

For the RD values, neither the linear nor quadratic model dominates, although the non-linear fit was slightly better. Where the quadratic model fit the data better, the regression curves show convex patterns with a nadir falling in the age studied, which means the diffusivity first slightly decreases and then increases after reaching the bottom (Fig 4A). The average nadir was 41.93±3.47 years for all data using the quadratic model. For linear models, the slopes of the lines were not significantly (P values are listed in Table 2) different from zero, which demonstrates no change of RD against age (Fig 4B). The RD values essentially did not show complementary changes in patterns with the FA values in the measured regions.

## Discussion

For the first time in a large cohort of healthy subjects, we studied subcortical WM changes associated with aging using an optimized high resolution DTI technique. The statistically decreased FA and increased RD values in the subcortical regions, especially in the anterior part of the brain, indicated demyelination associated with aging. We also found regions without significant changes, such as corpus callosum and internal capsules, which are mainly located in the central WM. Hsu et al also found that there was no significant age-related FA change in the splenium of corpus callosum[31]. The data in our study clearly showed an anterior-posterior gradient pattern in terms of WM deterioration, which is corroborated by traditional WM studies using low spatial resolutions.

In our study, ROIs of the WM of bilateral superior frontal gyri, bilateral precentral gyri, bilateral superior parietal lobes, and bilateral calcarine cortices belong to the subcortical WM. Unlike previous studies of subcortical WM [32, 33], we drew irregular ROIs only in the WM of the last branch of the cortex, respecting the border of the cortico-medullary junction. This means those ROIs comprise large quantities of dendrites and U-fibers of the cortex, which are important for local nerve networks. These U-fibers begin myelination early in gestation and often aren't completely myelinated until the third or fourth decade of life. As expected, FA showed an inverse relationship with age in the ROIs for the WM of bilateral superior frontal gyri, bilateral precentral gyri, and bilateral superior parietal lobes. Consistent with previous studies, the right prefrontal subcortical WM showed the most prominent effects of aging [34]. Larger age differences seen in FA in the frontal WM than the posterior WM are consistent with the anterior-posterior gradient of age related differences in WM [1]. The FA of occipital subcortical WM had no significant relationship with age, which has been previously reported [35]. However, RD of left occipital subcortical WM showed a concave pattern with quadratic regression, which demonstrated that RD might provide even more sensitive information for aging studies. A potential explanation for this finding could be that RD is less sensitive to image noise than FA, which is a second-order statistic of the diffusivity eigenvalues.

Age-related decrease of FA or increase of RD was also detected in the bilateral cingulum and the fornix, the associative fibers of the limbic system. It is of note that cingulum is also subcortical WM. Using quadratic fitting for the left cingulum and the fornix, it suggested that there was a turnover point around 40 years, after which the FA decreased and RD increased gradually. In the study by Hsu et al. [31], they reported that the turnover age for the global MD analysis was noted at 40.6±2.9 years old. Another study showed that white matter volume increased till age 43 and then declined thereafter [36]. The possible explanation may be that axonal arborization, increased myelination and WM growth contribute to the increased FA and decreased RD till the forth decade of life. After the turnover age, age-related alterations in the WM regions such as demyelination, deterioration and axonal loss may induce the opposite changes of DTI metrics.

However, no significant age-related effect was found for the FA of corpus callosum, or the posterior limbs of internal capsule in our study. Some studies reported no significant age-related FA change in the splenium of corpus callosum [31, 35]. Thin fibers have a predilection for loss, thus large-diameter axons and dense fibers might be the reason why the corpus callosum and internal capsules are relatively insensitive to the DTI measures.

Concordant age-related changes of FA decreases and RD increases may be interpreted to indicate processes of demyelination and Wallerian degeneration [37, 38]. Aging is associated with significant WM deterioration and this deterioration is assumed to be at least partly a consequence of myelin degeneration. Studies have reported several DTI measures related to aging, including FA, RD and AD. With the quadratic model, we found that for WM related to cognitive function, such as frontal subcortical WM and fornix, RD tended to increase after the 4^th^ decade, suggesting the onset of WM demyelination. However, FA in the subcortical regions showed a decrease from the very beginning using the linear model. Since aging is the result of loss of demyelinated nerve fibers as well as expansion of extracellular space [39], we speculate the age-related reduction in FA in young adulthood may arise from intravoxel increased interstitial fluid [12]. From this viewpoint, RD is superior to FA in the assessment of aging processes in that demyelination is more important for clinical evaluation. The effects of aging on WM and their impact on cognitive performance were stronger for radial RD than for axial diffusivity (AD), and RD is more sensitive to age-related changes in cognition [40]. The underlying mechanism may be that AD reflects molecular diffusivity parallel to axonal fibers while RD mainly reflects diffusivity perpendicular to axonal fibers. Thus theoretically, any increases in AD may reflect pathology of the axon itself, while RD may be more prominently correlated with demyelination, for example. According to the previous studies, myelin loss may induce changes in RD instead of AD [33]. This is also the reason that we assessed the changes of RD instead of AD to indicate the aging process.

Most previous studies utilized linear regression to investigate aging effects from DTI measures, whereas a few adopted quadratic regression. It was suggested that quadratic regression might be superior to linear regression in that it better fit data [31, 41]. We tested which model was better for FA and RD values. The linear model was adopted in our analysis when the 95% confidence interval for the quadratic coefficient in the quadratic model contains 0. Interestingly, for FA, the linear model was suitable for most ROIs, except the left cingulum and fornix, whereas the quadratic model was slightly more suitable for the fitting of RD data. To our knowledge, there is no consensus about the model selection. As the age range increases, the association between WM alterations and age may not necessarily be linear. It does not mean quadratic model would be the best either. In this study, we used a relatively flexible strategy to fit the data instead of using a fixed model for all data. From the data, we observed the quadratic model fit some data better, such as the fornix, while the linear model fit the left data better, such as left frontal subcortical WM. Also, using the quadratic model, the turnover age at around 40 years can be found. We noticed that different models were used in the same nuclei on both hemispheres sometimes, such as cingulum in Fig 3. One possible explanation could be that our cohort was still not large enough. Although the current data presented the difference mathematically, the result might be altered if more data are obtained. The accuracy of data interpretation definitely needs further investigation.

In this study, there were several limitations. First, we used a high in-plane spatial resolution, 0.94×0.94 mm^2^, which is four times higher than traditional studies using 2×2 mm^2^. However, in order to maintain sufficient SNR, the slice thickness, 4mm, was suboptimal which induced partial volume effects in the measurement along the slice direction, especially for occipital subcortical WM. We did not include temporal subcortical WM in the measurement because the WM of temporal gyri are mostly parallel to the axial plane. Compared with the commonly used resolution, such as 2.2×2.2×2.2 mm^3^, our technique indeed provides improved spatial resolution, which can provide images with less blurring and image distortion (increased sampling bandwidth along phase encoding direction), as well as suppression of the partial volume effect [20, 21]. The benefit in this study is that we can distinguish subcortical WM more easily from the surrounding tissue. Second, in order to minimize scanning time, we only used 6-direction DTI. Diffusion b factor was also limited to 800 sec/mm^2^. These parameters may affect the results according to the guidance by Bassar [42, 43]. Third, in this study, we found that data rejection was a must when images were acquired without cardiac triggering. However, after data rejection, the SNR was lowered. Different slice locations showed different SNR drops due to different rejection rates. This can be addressed by using data reacquisition techniques [44]. However, our MRI system does not yet support this. These issues are essentially related to the inherently low SNR of diffusion imaging. With the development of MRI technology, hopefully increased SNR or resolution can be achieved further. Fourth, this study only investigated the age-related changes among subjects with different ages (cross-section study) rather than changes associated with aging in each individual (longitudinal study). For longitudinal studies, the reliability of scanner systems must be maintained [45].

## Conclusion

By using multi-shot high resolution DTI, we have studied subcortical WM changes with aging. The results demonstrate that high resolution DTI can provide subtle information about the subcortical WM. The age-related decline of FA or increase of RD observed in subcortical WM may be indicative of a mild demyelination and a loss of myelinated axons. Compared with WM close to the central brain, subcortical WM has more prominent changes. As subcortical WM is involved very early in some neurodegenerative diseases, timely monitoring WM change is of importance in routine clinical practice and neuroimaging research.
